# Antimicrobial Peptides Controlling Resistant Bacteria in Animal Production

**DOI:** 10.3389/fmicb.2022.874153

**Published:** 2022-05-19

**Authors:** Gisele Rodrigues, Lucas Souza Santos, Octávio Luiz Franco

**Affiliations:** ^1^Centro de Análises Proteômicas e Bioquímicas, Programa de Pós-Graduação em Ciências Genômicas e Biotecnologia, Universidade Católica de Brasília, Brasília, Brazil; ^2^S-Inova Biotech, Programa de Pós-Graduação em Biotecnologia, Universidade Católica Dom Bosco, Campo Grande, Brazil

**Keywords:** antimicrobial resistance, growth promoters, antimicrobial peptides, livestock, feed supplementation

## Abstract

In the last few decades, antimicrobial resistance (AMR) has been a worldwide concern. The excessive use of antibiotics affects animal and human health. In the last few years, livestock production has used antibiotics as food supplementation. This massive use can be considered a principal factor in the accelerated development of genetic modifications in bacteria. These modifications are responsible for AMR and can be widespread to pathogenic and commensal bacteria. In addition, these antibiotic residues can be dispersed by water and sewer water systems, the contamination of soil and, water and plants, in addition, can be stocked in tissues such as muscle, milk, eggs, fat, and others. These residues can be spread to humans by the consumption of water or contaminated food. In addition, studies have demonstrated that antimicrobial resistance may be developed by vertical and horizontal gene transfer, producing a risk to public health. Hence, the World Health Organization in 2000 forbid the use of antibiotics for feed supplementation in livestock. In this context, to obtain safe food production, one of the potential substitutes for traditional antibiotics is the use of antimicrobial peptides (AMPs). In general, AMPs present anti-infective activity, and in some cases immune response. A limited number of AMP-based drugs are now available for use in animals and humans. This use is still not widespread due to a few problems like *in-vivo* effectiveness, stability, and high cost of production. This review will elucidate the different AMPs applications in animal diets, in an effort to generate safe food and control AMR.

## Introduction

In the last decades, antimicrobial resistance (AMR) has been a worldwide concern. The indiscriminate use of such drugs for a long time led to the formation of significant reservoirs of microorganisms with AMR genes in human and animal production (World Health Organization., 2014; Sharma et al., 2018).

The use of antimicrobials in animal feedstuff as therapeutic, metaphylactic, prophylactic, and growth promoter agents started in the year 1950, to boost food production (Krishnasamy et al., 2015; Woolhouse et al., 2015; Lagha et al., 2017; Magouras et al., 2017). The indiscriminate use of such drugs for a long time led to the formation of significant reservoirs of microorganisms with AMR genes in livestock production (World Health Organization., 2014; Sharma et al., 2018). Moreover, drug-resistant bacteria can disseminate in two ways: through direct contact with animals and humans or indirectly through the food chain, and contaminated environment (Soucy et al., 2015; Lagha et al., 2017; Magouras et al., 2017; Vidovic and Vidovic, 2020). In 2014, the World Health Organization (WHO), emphasized the abusive use of antibiotics in the treatment of infectious diseases can result in bacteria with genes resistant to these drugs (Brown et al., 2017) (Table 1). Hence, in 2000, the WHO Advisory Group on Integrated Surveillance of Antimicrobial Resistance (AGISAR) (2011) classified AMR as a global public health concern, recommending the eradication of the use of antibiotics for feed supplementation in livestock.

**Table 1:** Antimicrobial agents used in animals and humans.

**Antimicrobial class**	**Antibiotics**	**Animal use**	**Activity** **human**	**References**
Aminoglycosides	Gentamicin B	Therapeutic use for poultry and swine	Yes	Heuer et al. (2009)
	Lasalocid	AGP*	No	Heuer et al. (2009)
	Neomycin	Therapeutic use and AGP in cattle, swine, poultry and aquiculture	Yes	Jones and Ricke (2003)
	Streptomycin B	Feed supplementation for aquiculture	Yes	National Research Council. (1999)
Amphenicols	Florfenicol	Therapeutic use in cattle and swine	No	Dibner and Richards (2005)
	Carbomycin B	Feed supplementation for aquiculture	Yes	Bywater (2005)
Aminocoumarins	Novobiocin	Therapeutic use in bovine mastitis	Yes	Katsunuma et al. (2007)
Aminopenicillins	Amoxicillin, B ampicillin B	Therapeutic use in cattle, mastitis, swine, poultry and aquiculture	Yes	Aarestrup et al, 2007
Arsenicals	Roxarsone	AGP for poultry, swine and therapeutic use in swine	No	Witte (2000)
Beta-lactams	Procaine penicillin	AGP in poultry and swine	Yes	Witte (2000)
Cyclopolypeptides	Colistin	Feed supplementation for cattle, swine and broiler	Yes	Witte (2000)
Diaminopyrimidines	Ormetoprim	AGP and therapeutic use for poultry	No	Andleeb et al. (2020)
Elfamycins	Efrotomycin	AGP for swine	No	Bywater (2005)
Fluoroquinolones	Enrofloxacin B	Therapeutic use for cattle, swine	No	Bywater (2005)
	Flumequin B	Therapeutic use in aquaculture	No	Dibner and Richards (2005)
Glycopeptides	Ardacin	AGP for broilers	No	Arestrup et al. (2001)
	Avoparcin B	AGP	No	Arestrup et al. (2001)
Ionophores	Narasin	Feed supplementation and therapeutic use for poultry and AGP for cattle	No	Katsunuma et al. (2007)
	Maduramycin	Feed supplementation for poultry	No	Jones and Ricke (2003)
	Monensin	AGP in cattle and poultry	No	Jones and Ricke (2003)
	Salinomycin	AGP and therapeutic use for swine	No	Witte (2000)
Lincosamides	Lincomycin	Therapeutic use for poultry and swine	Rare	Heuer et al. (2009)
Macrolides	Macrolides	Therapeutic use for poultry	No	Bywater (2005)
	Tylosin B	AGP for swine and therapeutic use for mastitis	No	McEwen and Fedorka-Cray (2002)
	Oleandomycin B	AGP for swine and poultry	Yes	Andleeb et al. (2020)
	Erythromycin*b*	AGP in cattle, poultry, swine and therapeutic use in aquaculture	Yes	Dibner and Richards (2005)
	Spiramycin B	AGP for swine and therapeutic use in bovine mastitis	Yes	Witte (2000)
Nitrofurans	Furazolidone	Therapeutic use in aquaculture	Yes	Dibner and Richards (2005)
Orthosomysins	Avilamycin	AGP for broilers	No	Arestrup et al. (2001)
Phosphoglycolipids	Bambermycin	AGP	No	Butaye et al. (2003)
Pleuromutilins	Tiamulin	Therapeutic use and AGP for swine	No	McEwen and Fedorka-Cray (2002)
Polypeptides	Bacitracin/zinc bacitracin	AGP and therapeutic use in several livestock infections	Yes	Butaye et al. (2003)
Quinolones	Oxolinic acid B	Feed supplementation for aquiculture	No	Andleeb et al. (2020)
Quinoxalines	Carbadox	Therapeutic use in swine	No	Butaye et al. (2003)
	Olaquindox	AGP an therapeutic use in swine	No	Katsunuma et al. (2007)
Streptogramins	Pristinamycin	AGP	Yes	Andleeb et al. (2020)
	Virginiamycin	AGP for broilers	Yes	McEwen and Fedorka-Cray (2002)
Streptothricins	Nourseothricin	AGP for swine	No	Katsunuma et al. (2007)
Sulfonamides	Sulfonamides	Therapeutic use in aquiculture, and AGP in poultry and swine	Yes	National Research Council. (1999)
Tetracylines	Tetracyclines (oxy- and chlor-) B	AGP in cattle, poultry, swine and therapeutic use for livestock infection	Yes	National Research Council. (1999)

*Antimicrobial growth promoters.

In this sense, the use of alternative treatments such as phages therapy (Ferriol-González and Domingo-Calap, 2021; Loponte et al., 2021) and antimicrobial peptides treatment (Vieco-Saiz et al., 2019; Silveira et al., 2021) are considered to combat the advance of resistant microorganisms. In this review, we described information about antimicrobial peptides treatment.

Thus, the use of antimicrobial peptides (AMPs) suggests a possible alternative to traditional antibiotics, given their several modes of action, facility for degradation in nature, avoiding the accumulation, low resistance frequency, host immunity enhancement, and ability to neutralize the activity of many microbes (Jenssen et al., 2006; Zhao et al., 2016; Li et al., 2018). AMPs can be found in all organisms and demonstrated activity against several microorganisms even cancer cells (Saido-Sakanaka et al., 2004; Brogden, 2005; Hwang et al., 2011; Rodrigues G. et al., 2019; Rodrigues G. R. et al., 2019; Spohn et al., 2019; Vilas et al., 2019; Cardoso et al., 2020). Likewise, AMPs have sequences with variable structures, and mechanisms of action (Gomes et al., 2018; Spohn et al., 2019; Cardoso et al., 2020). Due to their cationic characteristics, AMPs may be capable of set electrostatic interactions with the external bacterial membrane, which is generally present negatively charged phospholipids (Hancock and Chapple, 1999; Shai, 2002). AMPs have the capacity to connect the outer membrane and act in the disturbed. In addition, they can also be translocated across the membrane and also react to internal targets (Hancock and Sahl, 2006). Furthermore, these peptides present the ability to stimulate the host's immune system indirectly (Hancock, 2001; Ward et al., 2013; Wang et al., 2016; Ageitos et al., 2017).

Therefore, this review will examine the different applications of AMPs supplemented in ruminants and non-ruminant feed, in an attempt to increase food production safety and control AMR.

## Antimicrobial Resistance and Environmental Problems

The discovery of penicillin represented an unprecedented milestone for modern medicine, transforming human history (Swann, 1983). Penicillin over the years has been collaborated to a massive reduction in mortality and caused an increase in life expectancy, besides offering essential support for invasive surgeries, and chemotherapy treatments (Blair and Piddock, 2009). Likewise, antibiotics also brought benefits to animal health when used as feed supplementation improving the growth and rentability of animal production (Cheng et al., 2014; Lhermie et al., 2017).

**Figure 1:**
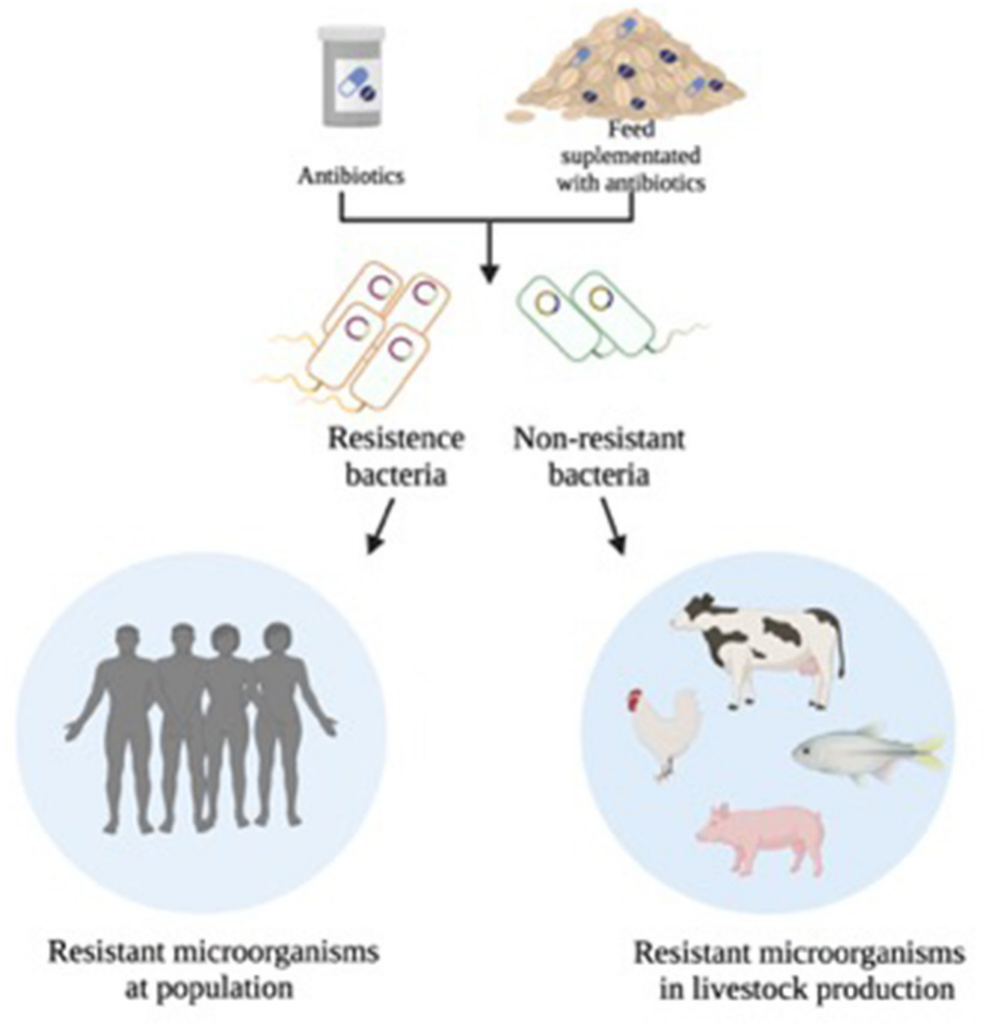
Representation of antibiotic-resistant bacteria in different ecosystems: antimicrobial usage may select the genes encoding resistance. Drug-resistant bacteria can spread when domestic animals receive antibiotics and develop antibiotic-resistant bacteria in their gastrointestinal tracts (GIT). This contamination can occur through humans, among vulnerable patients in hospitals or with contact with contaminated surfaces. These bacteria can be propagated in humans either through the food supply chain (meat and dairy products) or by direct animal contact. In addition, they can spread via water containing animal feces used for animal crops, or drug-resistant bacteria can remain on crops and be eaten. In this way, bacteria can remain in human and animal guts and spread in the community.

However, the antimicrobials used for animal food supplementation are the same as those administered as medicine for humans (World Health Organization., 2014; Sharma et al., 2018; Wu et al., 2018; Medina et al., 2020). The abusive use of antibiotics is the major factor in developing genetic modifications in bacteria. That is the main cause of antimicrobial resistance (AMR), which can be widespread in pathogenic and commensal bacteria (Thomas and Nielsen, 2005; Founou et al., 2016; Aslam et al., 2018; Li et al., 2018; Innes et al., 2020). AMR can be diffused into the food chain, by animal contact, or by environmental routes (Li et al., 2018; Scott et al., 2019) (Figure 1). Additionally, most of these drugs are not totally degraded in the body of animals and humans, and those residues are eliminated by excreted urine and feces, which then accumulate in soils, wastewater, manure causing profound, and complex impacts (Lim et al., 2013; Wu et al., 2014; Thanner et al., 2016; Li et al., 2018). Contact with or ingestion of antibiotic residues can give rise to several health problems, such as allergic hypersensitivity reactions, hepatotoxicity, nephropathy, mutagenicity, carcinogenicity, and antibiotic resistance (Mensah et al., 2014).

Presently, 700,000 annual worldwide death are associated with AMR, and the number of deaths in 2050 is estimated to reach 10 million (Aria and Murray, 2009; Munita and Arias, 2016; World-Health-Organisation [WHO], 2018; Ghosh et al., 2019). Considering all this information, the WHO recommended the suspension or elimination of the use of antimicrobial agents in animal feed supplementation. Following the recommendations of the WHO Advisory Group on Integrated Surveillance of Antimicrobial Resistance (AGISAR) (2011), countries of the European Union forbade feed supplementation with antibiotics in livestock production in 2006 (Magouras et al., 2017). In an attempt to standardize the measures to be taken and the information generated, surveillance and monitoring programs were created, advised by the WHO the OIE (OIE World Organisation for Animal Health., 2012), and the Food and Agriculture Organization (FAO) (FAO et al., 2018).

## Antimicrobial Peptides As An Alternative For Livestock Treatment

Livestock production is a sector that has expanded immensely, in an attempt to keep up with meat consumption. According to the FAO, cattle (including meat and dairy), pigs, and poultry together represent approximately 80% of the meat production (FAO, 2016, 2018). Current meat production is 200 million tons, and in 2050, this production will need to expand to 470 million tons, under current rates and predictions (Clifford et al., 2018). This rise causes concern regarding the quality of the meat produced (Vieco-Saiz et al., 2019), as accelerated production on large farms can cause health problems like weight loss, mastitis, and other infectious diseases (Krehbiel, 2013; Li et al., 2018; Sharma et al., 2018). Furthermore, farmers have been using antibiotics in their livestock production in an effort to prevent animal health problems, but the broad use of antimicrobials is one of the causes of the development of resistant microorganisms (World Health Organization., 2014; Sharma et al., 2018).

As described above AMPs, in general, demonstrated efficient activity against antimicrobial infection, due to the rapid action against pathogens, non-specific action, these result in a low resistance rate (Wimley and Hristova, 2011; Maria-Neto et al., 2015; Ageitos et al., 2017; Li et al., 2018). According to this, the overexposure of AMPs to the pathogens can generate the development of AMP-resistant strains.

### Antimicrobial Peptides Issues

AMPs demonstrated an efficient result acting as antimicrobial and immunomodulation activity. Despite this, AMPs may present some issues like bacterial resistance (Fry, 2018). This mechanism is unclear, but studies described that bacterial AMPs resistance cause alterations in membranes, cell walls, and cellular metabolism. In the case of membrane modification, bacteria can switch the AMP target, decreasing AMPs interactions with membrane components (Huhand and Kwon, 2011; Zucca et al., 2011). Also, these modifications can affect the permeability and fluidity of the membrane (Li et al., 2007; Otto, 2009).

Other resistance mechanisms result in a modification of bacterial ionic cell wall potential in specific interaction spots that can reduce the binding of antibiotic peptides (Henderson et al., 2014). In addition, AMPs activities against the bacteria could generate high metabolic stress levels like the production of proteases, modification of surface structures, and biofilm (Yeaman and Yount, 2003). Furthermore, AMPs also present problems related to high production costs compared with antibiotics, susceptible to enzymatic and pH degradation. AMPs that act in the gastrointestinal tract (GIT) occur in intestinal absorption, bioavailability, distribution, renal clearance, and peptide elimination (Fry, 2018; Meade et al., 2020).

In general, these issues can be avoided using computational strategies to overcome challenges associated with the high cost of production, the potency of AMPs, and reduce the rate of resistance, degradation, toxicity, and instability (Cardoso et al., 2020; Dijksteel et al., 2021). Another option is the use of multi-omics (including genomics, transcriptomics, and proteomics) which allows identifying a novel sequence of AMPs (Chen et al., 2019; Burgos-Toro et al., 2021).

Problems related above are responsible for the low number of peptides approved in a clinical trial because the efficiency of the results *in vitro* does not always the same as *in vivo*. Nevertheless, AMPs remain a great option to control microbial infections. Table 2 summarized some AMPs recently approved or in advanced clinical trials (Dijksteel et al., 2021).

**Table 2:** AMPs recently tested and approved by FDA.

**Peptide**	**Description**	**Target**	**Phase**	**Clinical Trial ID**	**Mechanism**	**References**
**Topical**						
PXL01	Analog of Lactoferrin	Postsurgical adhesions	II	NCT01022242	Immunomodulation	Edsfeldt et al., 2017
Wap-8294A2 (Lotilibcin)	Produced by Lysobacter species	Gram-positive bacteria	II/III		Membrane disruption	Itoh et al., 2018
Novexatin (NP213)	Cyclic Cationic peptide	Fungal nail infection	II	NCT02933879	Membrane disruption	Mercer et al., 2020
Melamine	Chimeric peptide	Contact lenses microbials	II/III		Membrane disruption	Yasir et al., 2019
Mel4	Derivative of melamine	Contact lenses microbials	II/III	ACTRN1261500072556	Membrane disruption	Yasir et al., 2020
D2A21	Synthetic peptide	Burn wound infections	III		Membrane disruption	Muchintala et al., 2020
Delmitide (RDP58)	Derivative of HLA	Inflammatory bowel disease	II		Immunomodulation	Travis et al., 2005
XOMA-629 (XMP-629)	Derivative of BPI	Impetigo/acne rosacea	III		Immunomodulation	Easton et al., 2009
PL-5	Synthetic peptide	Skin infections			Membrane disruption	Miyake et al., 2004
LTX-109	Synthetic tripeptide	MRSA/impetigo	I/II	NCT01803035; NCT01158235	Membrane disruption	Isaksson et al., 2011; Sivertsen et al., 2014
**Intravenous**						
hLF1-11	Fragment of human lactoferrin	Bacterial/fungal infections	I/II	NCT00430469	Membrane disruption/immunomodulation	Brouwer et al., 2018
EA-230	Oligopeptide	Sepsis	II	NCT03145220	Immunomodulation	van Groenendael, 2018
DPK-060	Derivative of Kininogen	Acute external otitis	II	NCT01447017	Membrane disruption/immunomodulation	Håkansson et al., 2019
Friulimicin	Cyclic lipopeptide	MRSA/pneumonia	I	NCT00492271	Membrane disruption	Schneider et al., 2009
Murepavadin (POL7080)	Analog of Protegrin	*P. aeruginosa, K. pneumoniae*	II	EUCTR2017-	Binding to LptD	Srinivas et al., 2010
IDR-1	Bactenecin	Infection prevention	I		Immunomodulation	Yu et al., 2009
Ghrelin	Endogenous peptide	Chronic respiratory infection	II	NCT00763477	Immunomodulation	Gualillo et al., 2003
PMX-30063 (Brilacidin)	Defensin mimetic	Acute bacterial skin infection	II	NCT01211470; NCT02052388	Membrane disruption/immunomodulation	Mensa et al., 2014
**Oral**						
Ramoplanin (NTI-851)	Glycolipodepsipeptide	*C. difficile*	III		Inhibition of cell wall synthesis	Fulco and Wenzel, 2006
SGX942 (Dusquetide)	Analog of IDR-1	Oral mucositis	III	NCT03237325	Immunomodulation	Kudrimoti et al., 2016
GSK1322322 (Lanopepden)	Synthetic hydrazide	Bacterial skin infection	II	NCT01209078	Peptide deformylase inhibitor	Peyrusson et al., 2015
NVB-302	Lantibiotic	*C. difficile*	I	ISRCTN40071144	Inhibition of cell wall synthesis	Crowther et al., 2013
Nisin bacteria	Polycyclic lantibiotic	Gram-positive		NCT02928042; NCT02467972	Depolarization of cell membrane	Prince et al., 2016

### AMP to Control Microbiota in Livestock Production

The microbiota profile relates to the growth performance of animals since the presence of specific groups of microorganisms promotes the absorption of nutrients inside the gastrointestinal tract (Yadav and Jha, 2019). The modulation of microbiota may also lead to the reduction of pathogenic species, decreasing the frequency and lethality of some diseases (Cheema et al., 2011; Wang et al., 2015b; Yadav and Jha, 2019).

Despite that, several diseases affected the livestock production causing intestinal mucosa inflammation, and diarrhea associated with morphological changes in the intestinal epithelium. These pathologies are caused by toxins produced by bacteria (Xiao et al., 2015). For decades, all diseases were treated using antibiotics which boosted the increase of antibiotic-resistant microorganisms. This increase in resistant bacteria in the animal microbiota has been demonstrated in resistome studies (Wang et al., 2021). Resitsome studies described the existence of a broad spectrum of antimicrobial resistance genes (ARGs) in the digestive tract of food-producing animals. The presence of ARGs is not necessarily associated with the direct use of antibiotics but can occur with the administration through feed or water or by injectable antimicrobials (Ma et al., 2021).

**Figure 2:**
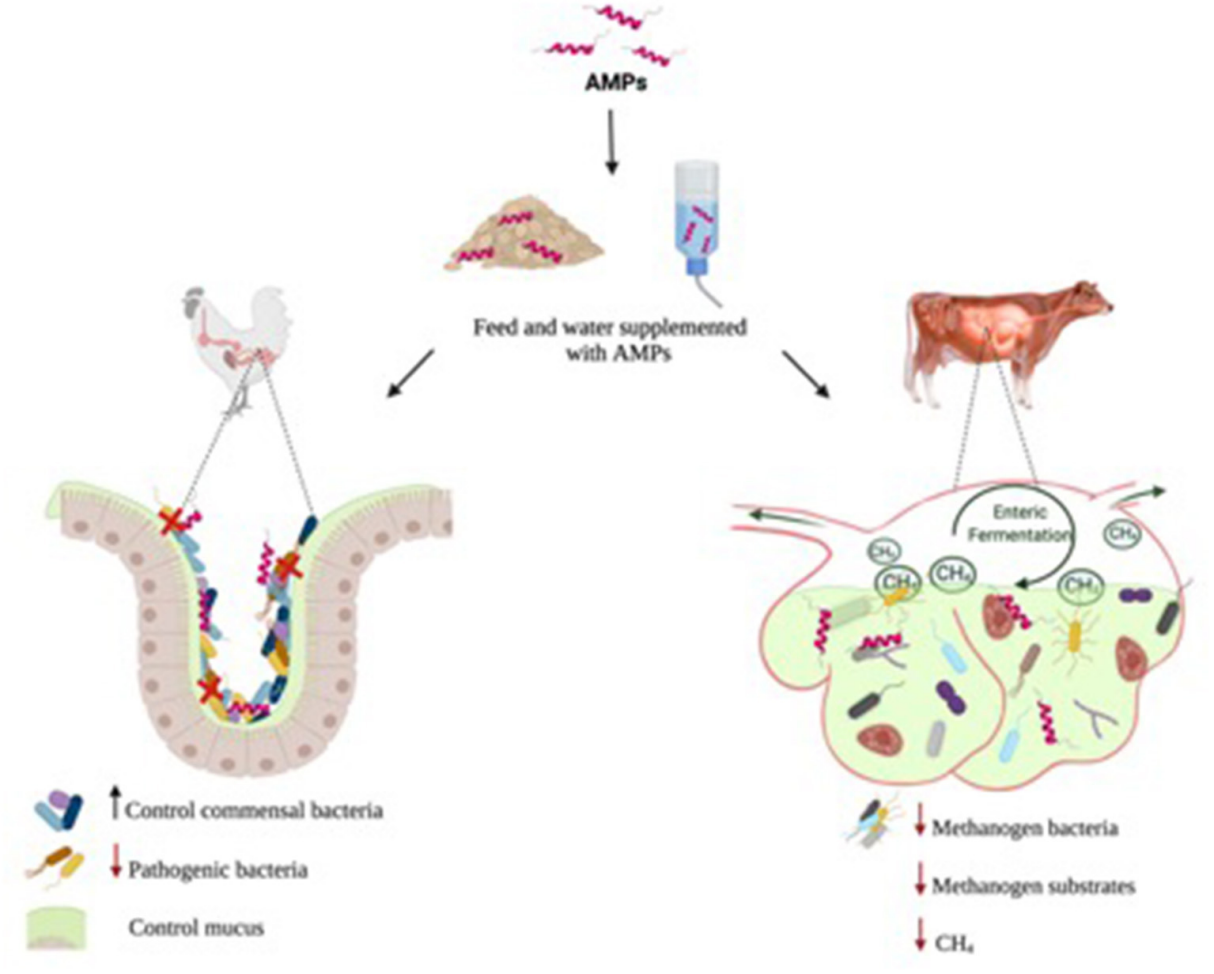
Antimicrobial peptides (AMPs) affects intestinal mucosa and rumen: AMP as growth promoters in poultry and swine intestinal can act altering the composition of the microbiota to reduce competition for nutrients, reduce pathogen, and control mucus. In rumen the AMPs as growth promoters acting reducing the methanogen bacteria and substrates. In addition, decrease the rate of methane (CH_4_) production and release.

In this context, the uses of AMPs utilization have demonstrated their ability to recover and maintain the GIT of animals by epithelial barrier integrity stabilization and by boosting intestinal epithelium colonization susceptibility (Murphy et al., 1993; Gallo et al., 1994; Podolsky, 2000; Tollin et al., 2003; Xiao et al., 2015) (Figure 2). Furthermore, some AMPs can act by inhibiting LPS-induced pro-inflammatory cytokine production, behaving as chemokines, or modulating the dendritic cell and T cell response (Mookherjee et al., 2006; Xiao et al., 2015).

Likewise, antibiotics have been used in ruminants with the goal to control the ruminal microbiota reducing losses during the enteric fermentation process. Moreover, ruminants are relevant sources of greenhouse gas (GHG) emissions (Eisler et al., 2014; Reisinger and Clark, 2018). The CH_4_ liberated for enteric fermentation suggests that 90% GHG is present in the atmosphere (Lan and Yang, 2019; Leahy et al., 2019). Other problems related to CH_4_ are the conversion to ammonia by rumen fermentation and its further excretion as urea in the urine can accumulate in the soil, and also cause groundwater pollution (Firkins et al., 2007) (Figure 2).

In this context, AMPs are used as a sustainable alternative to the rising production and mitigated contaminants. Peptides like LL32, Lpep 19-2.5, and NK2 derivatives of porcine NK-lysin have demonstrated activity against methanogenic archaeal strains and also observed in the control of rumen fermentation (Bang et al., 2012). This modulation can occur as an influence on electron flow, acting as the hydrogen acceptor to effectively compete with rumen methane production, or killing some nitrate-reducing Gram-positive bacteria (Bang et al., 2012; Shen et al., 2016, 2017; Varnava et al., 2017). Besides, some peptides use rumen microbiota to reduce amino acid deamination and methanogenesis, without having a negative impact on dry matter digestibility or volatile fatty acid production (Varnava et al., 2017). Additionally, the sheep feed supplemented with peptides showed a decrease in methane emission of 10% (Callaway et al., 1997; Shen et al., 2016). Thus, the use of AMPs in livestock can be an alternative method to solve problems with digestibility and microbiota, improving the sustainability of livestock production (Santoso et al., 2004; Sar et al., 2005; Wang et al., 2015a,b; Vieco-Saiz et al., 2019).

### AMPs Used as Growth Promoters

AMPs in feed supplementation have been extensively evaluated in several studies, and some characteristics are listed in Table 3. The peptide microcin J25 (MccJ25), a bacterial RNA polymerase inhibitor, increases the broilers' growth and attenuates the injuries to the intestine morphology caused by microbial infection. The application of MccJ25 in a range from 0.5 to 1.0 mg.kg^−1^ was able to reduce body weight loss by up to 70%, in comparison to 54.6% with antibiotic treatment (Wang et al., 2020). The recombinant cecropin A-D-Asn is formed by a chimeric peptide, from cecropin A, and cecropin D C-termini. Moreover, asparagine residue was added and amidated in C-terminus. The inclusion of 6 mg/kg^−1^ of the peptide to the basal feed of broilers boosts by 20% the weight when compared with feed without peptide addition (Wen and He, 2012).

**Table 3:** AMPs using livestock production.

**AMP**	**Source**	**Activity**	**Target bacteria**	**Animal**	**References**
Microcin J25	*E. coli*	Immune Regulation, and Intestinal Microbiota	*Escherichia coli, Salmonella* CVCC519	Broiler	Wang et al. (2020) and Iseppi et al. (2021)
Pediocin A	*Pediococcus pentosaceus*	Dietary supplementation	*Clostridium perfringens*	Broilers	Grilli et al. (2009) and Hernández-González et al. (2021)
Gallinacin-6	*Gallus gallus domesticus*	Antimicrobial	*Campylobacter jejuni, Salmonella enterica, Clostridium perfringens, E. coli*	Broilers	van Dijk et al. (2007)
Plectasin	*Pseudoplectania nigrella*	Dietary supplementation		Broilers	Ma et al. (2019)
RSRP	*Oryctolagus cuniculus*—sacculus rotundus	Dietary supplementation Intestinal mucosal immune responses	Reducing the viable counts of *E. coli*	Broilers	Liu et al. (2008)
Lactoferrin (bLf)	*Bos taurus*	Dietary supplementation Intestinal mucosal	Reducing the total viable counts of *E. coli* and Salmonella	Broilers	Tang et al. (2008); Messaoudi et al. (2012); Aguirre et al. (2015)
SMXD51	*Lactobacillus salivarius*	Intestinal Microbiota	*Campylobacter jejuni*	Poultry	Cao et al. (2007); Ceotto-Vigoder et al. (2016)
BT	*Brevibacillus texasporus*	Dietary supplementation Intestinal mucosal	*Salmonella enterica serovar Enteritidis*.	Neonatal poultry	Kogut et al. (2013)
Nissin*	*Lactococcus sp. Streptococcus sp*.	Food preservation; Antimicrobial	*E. coli, Staphylococcus aureus, Streptococcus agalactiae, S. dysagalactiae, S. uberis S. aureus* biofilm	Cattle	Santoso et al. (2004); Sar et al. (2005); Cao et al. (2007); Ceotto-Vigoder et al. (2016); Shen et al. (2016, 2017); Shin et al. (2016); Hernández-González et al. (2021)
Lysostaphin	*Staphylococcus sp*.	Antimicrobial	*S. aureus* biofilm	Cattle	Ceotto-Vigoder et al. (2016)
AP-CECT712	*Enterococcus faecalis*	Antimicrobia	*S. aureus, S. dysgalactiae*, S. *uberis*, S. *agalactia*	Cattle	Sparo et al. (2009)
Colicin	*E. coli*	Antimicrobial	*E. coli*	Swine	Stahl et al. (2004); Cutler et al. (2007)
Porcine (pBD-1)	Porcine blood	Antimicrobial, immune responses	*Bordetella pertussis*	Newborn Piglets	Elahi et al. (2006)
Cathelicidin-BF (C-BF)	*Bungarus fascia*	Intestinal immune responses		Weanling piglets	Wang et al. (2008); Yi et al. (2015)

*Commercial use—FDA liberation.

Pediocin A was administrated in poultry food and demonstrated efficient results as a growth promoter (Daeschel and Klaenhammer, 1985). A similar result with a gain of body weight was described using the combination of bacteriocins (divercin AS7 and nisin) as a food additive for broilers (Józefiak et al., 2013; Hernández-González et al., 2021). *In vivo* studies have shown AMPs also improve growth performance and digestive capacity in poultry and pigs (Wang et al., 2016). The use of AMP-A3 and AMP-P5 (both derived from the amino acid substitution of the *Helicobacter pylori* HP and the cecropin-magainin2 fusion, respectively), can raise the F:G ratio of weanling pigs and broilers, with additional benefits concerning nutrient uptake and intestinal morphology. The AMP-A3 (90 mg.kg^−1^) and AMP-P5 (60 mg.kg^−1)^, display effective results showing elevated weight gain and reduced intestinal damage (Yoon et al., 2012, 2013, 2014; Choi et al., 2013a,b).

Ren et al. (2019) demonstrated the use of the recombinant swine defensin PBD-mI with a molecular mass of 5.4 kDa, and LUC-n with a molecular mass of 21.18 kDa, in 18 4-month-old Chuanzhong black goats. The animals were split into three groups (basal diet; basal diet + 2g AMP/goat/day; basal diet + 3g AMP/goat/day), and rumen fluid was collected and analyzed. Dietary supplementation with both AMPs demonstrated that the goats enhanced rumen microbiota diversity, updated ruminal fermentation, improved efficiency of food usage, and boosted growth performance. Although studies demonstrated positive results of AMPs in feed supplementation for poultry and pigs, the same is not observed for ruminants.

### Use of AMPs to Control Infectious Disease

AMPs present an important role in controlling infection disease and the immunity system of non-ruminants maintaining (Hernández-González et al., 2021). Daneshmand et al. (2019), demonstrated that the use of a lactoferrin-derived peptide, cLF36 utilization can diminish infection by modulating the expression of cytokines IL-2 and IL-6 and mucine in broilers challenged with enterotoxigenic *Escherichia coli*. Adding 20 mg.kg^−1^ of cLF36 in feed reduced the population of *E. coli* and *Clostridium* spp. by 25% and 20%, respectively. Besides, the number of beneficial *Lactobacillus spp*. and *Bifidobacterium spp*. increased by up to 36%. Moreover, sublacin, a peptide obtained from *Bacillus*, may decrease harmful bacteria without causing any change in the *Lactobacillus* community. The peptide was supplemented with water (5.76 mg. L^−1^) (Wang et al., 2015a,b).

Another host defense peptide, ß-defensin-1 (pDB-1), has potential veterinary application. This peptide has shown its expression in the respiratory tract of old pigs, and demonstrated to be resistant against the infection of the respiratory pathogen *Bordetella pertussis*. Otherwise, newborn piglets do not seem to have pDB-1, and are susceptible to the disease. Thus, the application of 500 μg of tpDB-1 to the respiratory tract of these piglets was able to totally inhibit clinical symptoms (Elahi et al., 2006).

Furthermore, the peptide C-BF, which originates from *Bungarus fasciatus* venom, also demonstrated beneficial results in controlling bacterial disease in animal production (Elahi et al., 2006). C-BF used 0.5 mg.kg^−1^ in piglets via intraperitoneal application, and the peptide minimized the inflammatory molecule's TNF-α and IL-6. The level of cell apoptosis and intestinal barrier damage caused by bacterial lipopolysaccharide also decreased (Zhang et al., 2017). S100A8 and S100A9 showed beneficial results against ruminant infections. These peptides reduced uterine inflammation (which appears after calving in association with bacterial contamination) and modulated the early endometrial response against infection in Holstein–Friesian cows (Swangchan-Uthai et al., 2013).

Another application for AMPs is in aquaculture, a sector which dedicated to producing aquatic plants and animals, with a recent growth rate higher than any other land-based livestock (Gyan et al., 2020; León et al., 2020). *In vitro* study demonstrated high efficacy of synthetic peptides (frog caerin1.1, European sea bass dicentracin (Dic) and NK-lysin peptides (NKLPs) and tongue sole NKLP27) against viral fish pathogens, such as nodavirus (NNV), viral septicemia hemorrhagic virus (VHSV), infectious pancreatic necrosis virus (IPNV) and spring viremia carp virus (SVCV) (León et al., 2020).

In addition, Table 2 summarized many AMPs used in veterinary treatment with an efficient result.

## Application of Amps In Different Sectors

AMPs presented beneficial results in the control of microbial infections and in food supplementation. However, peptides have different functions in the food industry (Bemena et al., 2014; Rai et al., 2016), and artificial breeding in livestock (Schulze et al., 2014, 2020; Speck et al., 2014; Shaoyong et al., 2019).

The food industry normally uses nitrites and sulfur dioxide (chemical preservatives), which can cause negative effects on human health and the nutritional level of food (Bemena et al., 2014). Recently, AMPs have been used instead, to maintain the properties of the food without modifying quality, besides not being harmful (Wang et al., 2016). The lactic acid bacteria are a good example because they are recognized as safe by the Food and Drug Administration, and are extensively used in human and animal food as a preservative, and to control pathogenic and spoilage bacteria (Rai et al., 2016; Venegas-Ortega et al., 2019; Iseppi et al., 2021).

AMPs are also being studied and applied to semen preservation in the artificial breeding process. A recent study used two synthetics cyclic hexapeptides, c-WFW and c-WWW, and magainin II (MK5E). These peptides were tested for boar semen preservation, indicating that cyclic hexapeptides can be promising candidates, due to proteolytic stability, capacity to control bacterial proliferation, and synergistic interaction with conventional antibiotics. The peptide ε-PL also showed effective results at a low concentration (0.16 g. L^−1^), suggesting that it could be a possible substitute for gentamicin to enhance sperm quality parameters, sperm capacitation, and *in vitro* fertilization by reducing bacterial concentrations (Shaoyong et al., 2019).

## Concluding Remarks and Prospects

The excessive use of antibiotics as a growth promoter in livestock causes microbial resistance, which is associated with increased consumption of animal protein, while production has difficulties in keeping up with this demand (Eisler et al., 2014).

Hence, various countries prohibited antibiotics in animal supplementation, thus stimulating the expansion of research to sustainable approaches (Wang et al., 2016; Li et al., 2018; Leahy et al., 2019). Besides that, livestock products have faced challenges such as reduced productivity, loss of biodiversity, rising GHG emissions, sick animals, and diseases that can cause human illness (Grace et al., 2012; Michalk et al., 2019). Thus, sustainable animal production is the next step to increasing healthy livestock production and at the same time reducing environmental impacts (Kemp and Michalk, 2011; Godfray and Garnett, 2015; Vidovic and Vidovic, 2020).

Herein, we demonstrated positive results in the use of AMPs, which have shown to be promising in controlling microbial infection (Stahl et al., 2004; Ceotto-Vigoder et al., 2016), and methane gas emissions (Santoso et al., 2004; Sar et al., 2005), while also providing in-feed supplementation (Wang et al., 2008, 2016; Ren et al., 2019).

In this context, synthetic biology (SB) is an approach responsible for improving or completely creating systems and organisms, providing novel diagnostic tools, and enabling the economic production of new therapeutics drugs (Weber and Fussenegger, 2012; Takano and Breitling, 2014). SB has the skills to produce antibiotic drug advances, using different approaches like synthetic gene circuits (Weber et al., 2008) and protein engineering (King et al., 2016). It can foster the development of new drugs using faster and more efficient protocols, allowing the development of more accessible medicines that demonstrate greater precision (Noel, 2010; Jakobus et al., 2012). The rational design seeks to improve AMP sequence optimization and enhance biological activities, aiming to develop new drugs with high specificity against microorganisms and a reduction in adverse effects (Porto et al., 2012; Cardoso et al., 2020). In this context, computational tools like quantitative structure-activity relationship (QSAR), *de novo*, linguistic, pattern insertion, and evolutionary/genetic algorithms are very useful in designing AMP variants (Chen and Bahar, 2004; Loose et al., 2006; Hiss et al., 2010; Mitchell, 2014; Torres and De La Fuente-Nunez, 2019). In addition, these computational tools can be used separately or in association to construct novel peptide-based drug candidates (Cardoso et al., 2020).

In addition, AMPs can be used associated with nanoparticles (NPs) (Sharma et al., 2018). They could have several shapes and formulations (e.g., nitric oxide-releasing nanoparticles, chitosan-containing, and metal-containing nanoparticles) (Huhand and Kwon, 2011; Pelgrift and Friedman, 2013), and delivery systems, such as microencapsulation (Ganesh and Hettiarachchy, 2016; Kaikabo et al., 2016; Suresh et al., 2018), improving the bacterial control system. NPs can boost the effectiveness in the treatment of infectious diseases, besides protecting the peptide from degradation in the physiological environment (Rodrigues G. et al., 2019; Rodrigues G. R. et al., 2019). These tools are able to produce new drugs with fewer side effects, low costs, and with ability to abolish or control infectious diseases.

Different studies have been executed in the search for AMPs with anti-infective activities, but it is essential that these studies proceed to *in vivo* models and also to clinical trials.

All alternate strategies suggested can be successfully implemented with the prudent use of antibiotics, and strengthen the supervision associated with policies and regulation of use. These steps will allow farmers and veterinarians to prescribe treatment options for livestock production without causing chain effects. Thus, the use of AMPs in livestock allows the safe production of quality food, contributing to the maximization of agricultural output in a sustainable and economically satisfactory way.

## Author Contributions

All authors listed have made a substantial, direct, and intellectual contribution to the work and approved it for publication.

## Funding

This work was supported by grants from Fundação de Apoio à Pesquisa do Distrito Federal (FAPDF), Coordenação de Aperfeiçoamento de Pessoal de Nível Superior (CAPES) (MC 88887.351521/2019-00), Conselho Nacional de Desenvolvimento e Tecnológico (CNPq), and Fundação de Apoio ao Desenvolvimento do Ensino, Ciência e Tecnologia do Estado de Mato Grosso do Sul (FUNDECT), Brazil.

## Conflict of Interest

The authors declare that the research was conducted in the absence of any commercial or financial relationships that could be construed as a potential conflict of interest.

## Publisher's Note

All claims expressed in this article are solely those of the authors and do not necessarily represent those of their affiliated organizations, or those of the publisher, the editors and the reviewers. Any product that may be evaluated in this article, or claim that may be made by its manufacturer, is not guaranteed or endorsed by the publisher.
